# Efficacy and safety of perioperative appliance of sunitinib in patients with metastatic or advanced renal cell carcinoma: A systematic review and meta-analysis

**DOI:** 10.1097/MD.0000000000015424

**Published:** 2019-05-17

**Authors:** Hongyu Jin, Jing Zhang, Kai Shen, Jianqi Hao, Yuying Feng, Chi Yuan, Yuqi Zhu, Xuelei Ma

**Affiliations:** a Department of Biotherapy and Cancer Center, State Key Laboratory of Biotherapy, West China Hospital; b West China School of Medicine; c Department of Liver Surgery, Liver Transplantation Center, West China Hospital, Sichuan University, Chengdu 610041, China.

**Keywords:** adverse effects, efficacy, perioperative use of sunitinib, safety

## Abstract

**Background:**

The aim of this systematic review and meta-analysis is to comprehensively evaluate the efficacy and safety of the perioperative use of sunitinib in patients with metastatic and advanced renal cell carcinoma (RCC).

**Materials and methods:**

We searched authenticated databases for related clinical studies. The baseline characteristics, parameters concerning the efficacy and safety of the perioperative use of sunitinib were extracted for subsequent comprehensive analysis. The parameters which reflected the efficacy and safety as overall survival (OS), progression-free survival (PFS), occurrence rate of all-grade and grade ≥3 adverse effects (AEs) were carefully pooled using comprehensive meta-analysis.

**Results:**

We finally recruited 411 patients from 14 eligible studies. We found proteinuria (75.0%, 95% CI 62.1%–84.6%), anemia (71.6%, 95% CI 60.9%–80.3%), athesia (60.0%, 95% CI 40.3%–77.0%), pause symptoms (59.2%, 95% CI 49.2%–68.4%), arterial hypertension (53.1%, 95% CI 43.2%–62.7%), and thrombocytopenia (52.5%, 95% CI 44.8%–60.0%) to be the most common all-grade AEs. And arterial hypertension, athesia, cutaneous toxicity, hypophosphatemia, leukopenia, pain, pause syndrome, renal dysfunction, and thrombocytopenia were the most common types of grade ≥3 AEs. In addition, objective response rate (ORR) of sunitinib to both the original and metastatic tumor sites increased with the use of sunitinib, so did the OS and PFS.

**Conclusion:**

Common all-grade and grade ≥3 AEs were carefully monitored. The perioperative use of sunitinib showed superior ORR, OS, and PFS rates. Nevertheless, more studies are required to further verify these findings.

## 1 Introduction

Renal cell carcinoma (RCC) is reported to cause approximately 78,000 deaths among 150,000 people attacked worldwide. Particularly, the global mortality doubled from 1985 to 2000.^[1,2]^ Notably, rapid and unexpected progress along with invasiveness enhancement are often observed in RCC.^[3]^ Among all malignant progress, direct metastasis through potential cavities in the abdomen, pernicious metastasis through blood vessels and the formation of venous thrombus into the right atrial system are most widely discussed.^[4,5]^ Unfortunately, effective therapy for metastatic and advanced RCC is still limited.^[6]^ So far, surgical removal and traditional therapeutics are still the widest applied strategies for metastatic and advanced RCC, especially in patients with intravenous tumor thrombus. However, surgical intervention to remove tumor thrombus is often challenging since it requires sternotomy and optional cardiac arrest assisted by extracorporeal circulation.^[7]^ Therefore, adjuvant therapy with surgery and chemotherapy should be explored and investigated.

Sunitinib is an orally taken agent which is a multi-targeted tyrosine kinase inhibitor (TKIs) including vascular endothelial growth factor receptors (VEGFRs), like VEGFRs (VEGFR-1, VEGFR-2, and VEGFR-3) and c-Kit, etc, which are the mostly identified element in RCC pathogenesis and progress.^[8]^ RCC is driven by angiogenesis and early hypoxia, in which angiogenesis is proved to be an independent prognostic factor.^[9,10]^ Therefore, the neoadjuvant therapy combining the use of sunitinib and surgery has been put forward in the treatment of metastatic and advanced RCC. Up to now, dozens of studies including two famous landmark trials have demonstrated the role of the combining therapy in the alleviation and downstaging in patients with metastatic and advanced RCC,^[11,12]^ claiming that the particular preoperative and intraoperative use of sunitinib is responsible for the decrease in both the volume and downstaging of the original and metastatic tumor as well as the tumor thrombus.^[13,14]^ Moreover, survival analysis by other researches manifested by overall survival (OS) and progression-free survival (PFS) have also testified its efficacy.^[15,16]^

However, some studies have also pointed out the inefficacy and several safety concerns related to perioperative appliance of sunitinib.^[17]^ Accordingly, sunitinib related hand and foot syndrome, malaise in the digestive tract, several abnormalities in the concentration of blood cells are regarded as major AEs and health troubling issues of sunitinib.^[18]^ Therefore, in order to comprehensively analyze the therapeutic efficacy and safety issues of perioperative use of sunitinib in patients with metastatic and advanced RCC, we performed this systematic review and meta-analysis based on valuable and trustworthy studies worldwide.

## 2 Materials and methods

### 2.1 Search strategy

Following the guidelines for performing meta-analysis, we searched authenticated databases including PubMed/Medline, Web of Science, Cochrane Library, ClinicalTrials.gov (http://www.ClinicalTrials.gov), China National Knowledge Infrastructure (CNKI) for related articles published from January 2008 to May 2018. Articles we primarily searched were subsequently screened for its relevancy and availability. No language restriction was used.

### 2.2 Article selection

Two independent reviewers participated in the screening process who analyzed the full texts and performed quality and relevancy assessment. The inclusion criteria included: first, reported at least either indicators for survival analysis or data concerning the AEs; and second, randomized controlled trials and any observational design, including cross-sectional, case-control, and cohort designs. Subsequently, we performed a blinded cross-check to detect underlying discrepancies. If a discrepancy was detected, a third reviewer was assigned to adjudicate the conflict. The identification, inclusion and exclusion of studies were conducted according to reporting items for systematic reviews and meta-analysis (PRISMA) guidelines.

Two experienced investigators independently analyzed relevant articles for parameters concerning the efficacy and safety of perioperative sunitinib appliance. The discrepancies were discussed and resolved subsequently. The key parameters included OS in 10, 20, 30, and 40 months, PFS in 10, 20, and 30 months, objective response rate (ORR), stable disease (SD) rate, progressive disease (PD) rate, median OS and PFS, types of AEs and their occurrence rates, etc. In addition, baseline characteristics of the articles including title, first author, nationality, department, ethnicity, study design, sex and median age of the patients, and enrollment year were also carefully extracted.

### 2.3 Statistical analysis

The occurrence rate of AEs, including AEs of all grades and of grades ≥3 AEs as well as their 95% confidential interval (CIs) were calculated based on data collected from these single-arm trials. All the analyses and calculations mentioned above were conducted using comprehensive meta-analysis (CMA) (Biostat, Englewood, NJ).

The study was approved by the Ethics Committee of West China Hospital, Sichuan University (Chengdu, China).

### 2.4 Quality assessment

**Figure 2:**
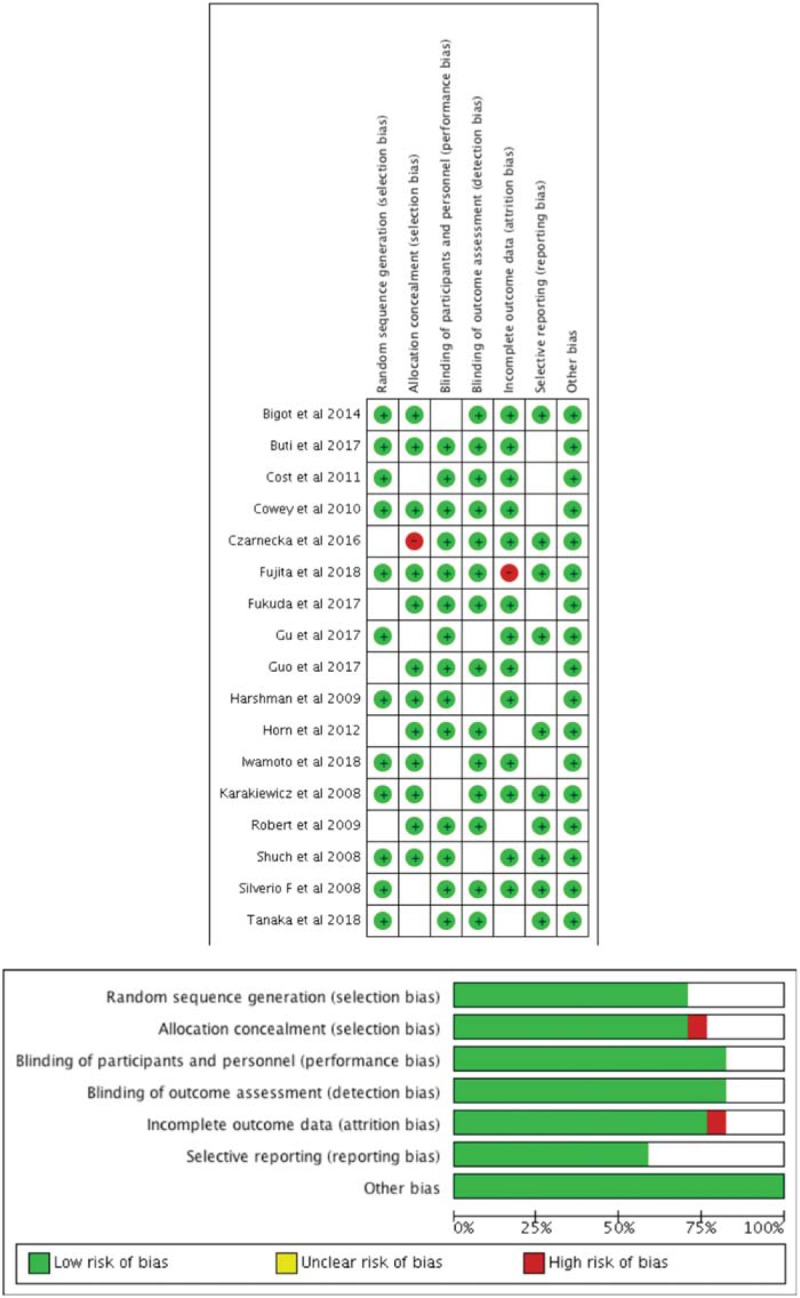
Quality evaluation based on the Quadas-2 evaluating tool.

Standard quality evaluation of the included studies was performed based on the Quadas-2 tool (Fig. 2).^[19]^ Particularly, the risk of bias was obtained by RevMan 5.3 (The Cochrane Collaboration). The articles were evaluated in the following processes: sequence generation (selection bias), allocation concealment (selection bias), blinding of participants and personnel (performance bias), blinding of outcome assessment (detection bias), incomplete outcome data (attrition bias), selective reporting (reporting bias), and others. According to Quadas-2 evaluating systems, the included studies were ultimately defined as reliable. Accordingly, the method used to select patients may have contributed to bias.

## 3 Results

### 3.1 Evidence acquisition

**Figure 1:**
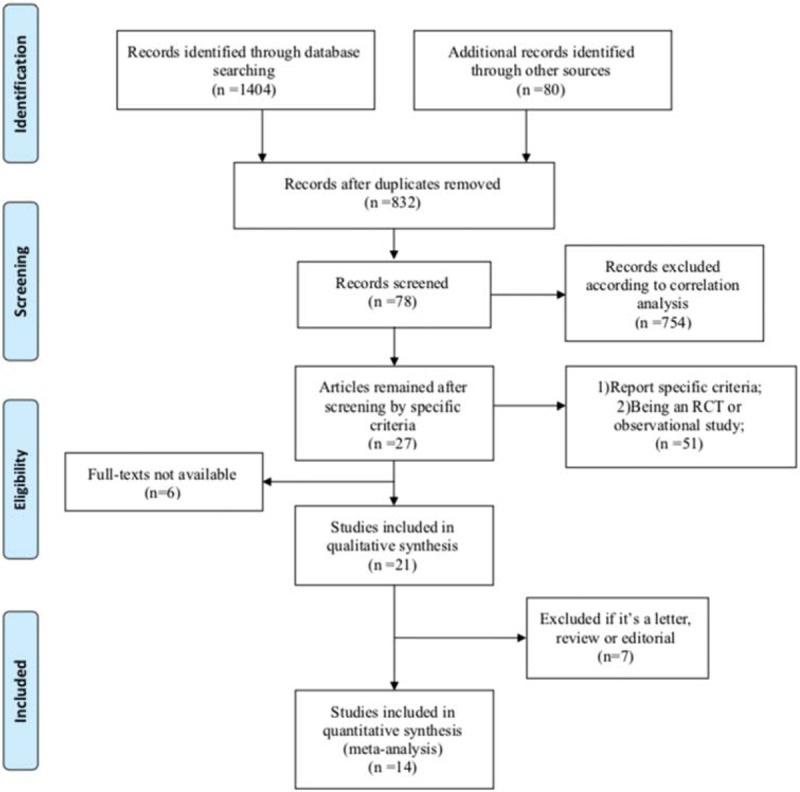
PRISMA flow diagram of the article selection process.

The primary search through the eight authenticated and other sites yielded 1484 studies. Eight hundred thirty-two (832) studies remained after removal of obvious duplicates. Next, a meticulous correlation analysis was performed for availability and eligibility, in which process 754 studies were excluded. The remaining 78 studies were carefully considered and were excluded when failing to meet the two significant criteria mentioned above, after which only 27 studies left. Of them, 6 studies were further removed since the full-texts were unavailable; 7 studies were not considered for this meta-analysis because they were reviews, letters, and editorials. Finally, we recruited 14 eligible, reliable studies into this meta-analysis. The identification, inclusion, and exclusion of studies were conducted according to PRISMA guidelines. Figure 1 shows the PRISMA flow diagram of the article selection process.

### 3.2 Characteristics of the included studies

After a carefully planned screening process, 14 studies were eventually considered for this meta-analysis. All the included studies were published between 2008 and 2018 with eight of them published after 2014. The 14 studies totally recruited 411 patients with metastatic RCC, advanced RCC or RCC with venous tumor thrombus from Asian (n = 183), Europe (n = 211), and North America (n = 27). Patients recruited took sunitinib before, during or after the operation. Twelve studies carefully recorded the change of the tumor size or grade, three studies provided the OS and PFS curves and six studies recorded the incidence of AEs. The detailed information and baseline characteristics of each study we included was shown in Table 1.

**Table 1:**
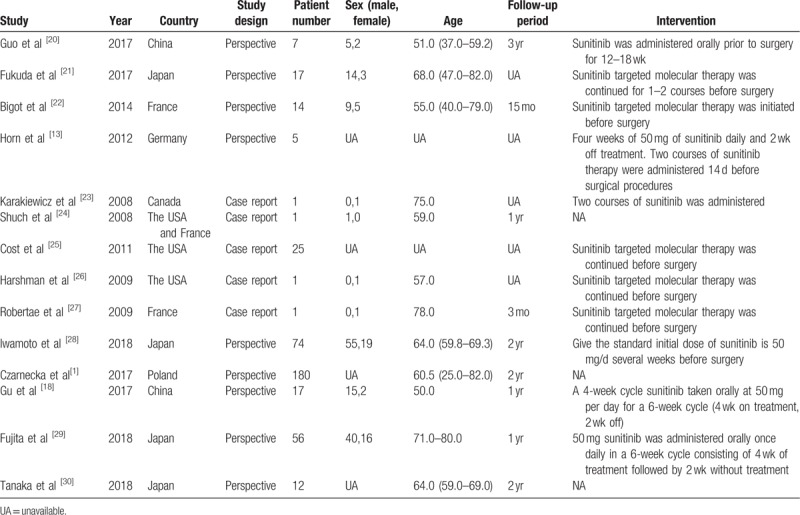
Basic characteristics of the included studies.

### 3.3 Safety analysis of the perioperative use of sunitinib

**Figure 3:**
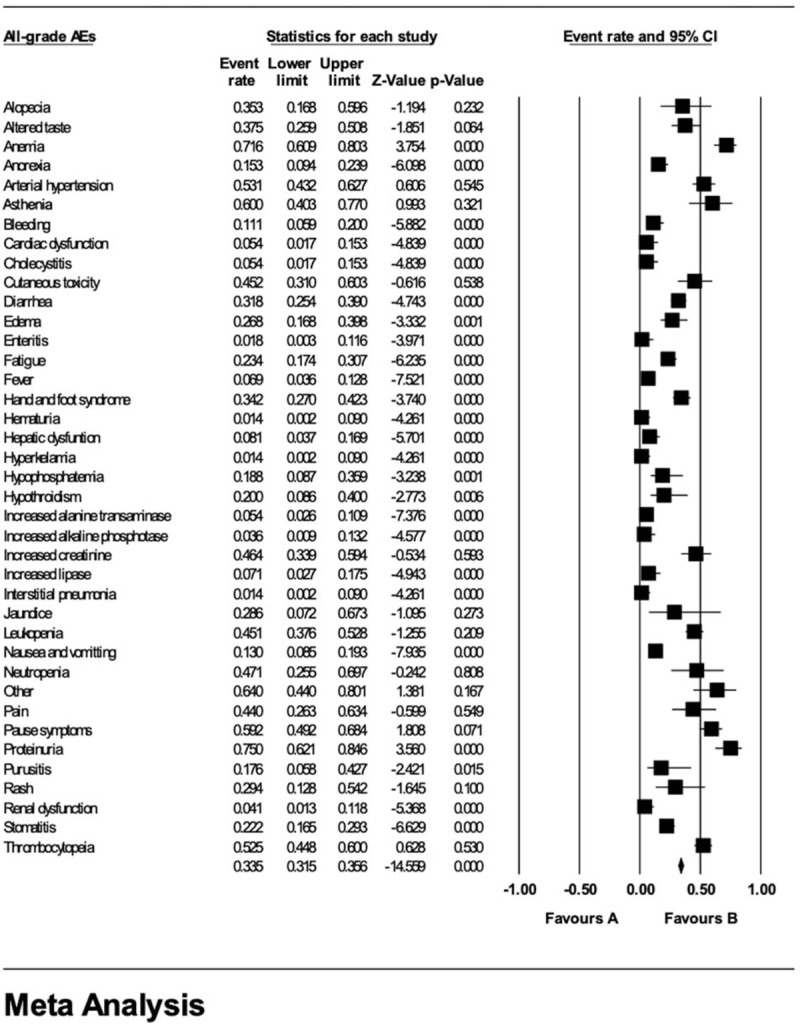
Pooled rates of all-grade AEs.

**Figure 4:**
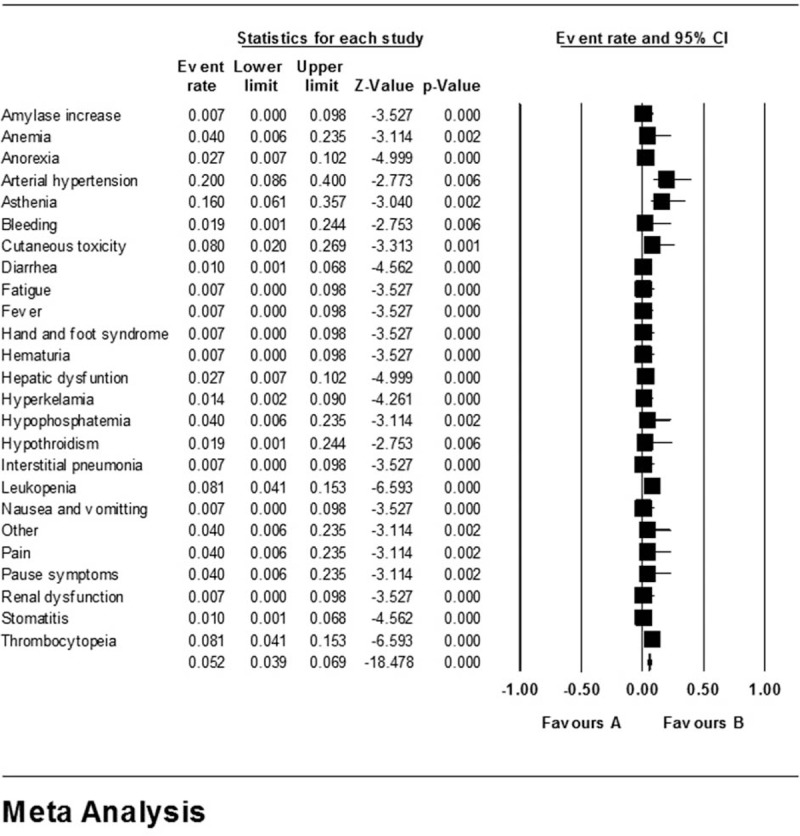
Pooled rates of grade ≥3 AEs.

In order to objectively calculate the rates of all-grade and grade ≥3 AEs, the related data in the eligible studies were extracted and pooled (Figs. 3 and 4). Statistically, within all-grade AEs, proteinuria was found to maintain the highest rate (75.0%, 95% CI 62.1%–84.6%), followed by anemia (71.6%, 95% CI 60.9%–80.3%), asthenia (60.0%, 95% CI 40.3%–77.0%), pause symptoms (59.2%, 95% CI 49.2%–68.4%), arterial hypertension (53.1%, 95% CI 43.2%–62.7%), and thrombocytopenia (52.5%, 95% CI 44.8%–60.0%). However, proteinuria was only observed in one study by Tetsuo Fujita^[29]^ which involved 36 patients, thus there might be potential bias regarding this AE. Other common AEs included neutropenia (47.1%, 95% CI 25.5%–69.7%), increased creatinine (46.4%, 95% CI 33.9%–59.4%), pain (53.1%, 95% CI 26.3%–63.4%). There were also several rare AEs including anorexia (15.3%, 95% CI 9.4%–23.9%), bleeding (11.1%, 95% CI 5.9%–20.0%), enteritis (1.8%, 95% CI 0.3%–11.6%), hematuria (1.4%, 95% CI 0.2%–9.0%), etc, as shown in Figures 3 and 4.

Among grade ≥3 AEs, arterial hypertension maintained the highest occurrence rate which was (20.0%, 95% CI 8.6%–40.0%). Other grade ≥3 AEs which had an occurrence rate over 4.0% included asthenia (16.0%, 95% CI 6.1%–35.7%), leukopenia (8.1%, 95% CI 4.1%–15.3%), thrombocytopenia (8.1%, 95% CI 4.1%–15.3%), cutaneous toxicity (8.0%, 95% CI 2.0%–26.9%), hypophosphatemia (4.0%, 95% CI 0.6%–23.5%), pain (4.0%, 95% CI 0.6%–23.5%), and pause syndrome (4.0%, 95% CI 0.6%–23.5%). Others included amylase increase, anemia, anorexia, bleeding, diarrhea, fatigue, fever, etc.

**Figure 5:**
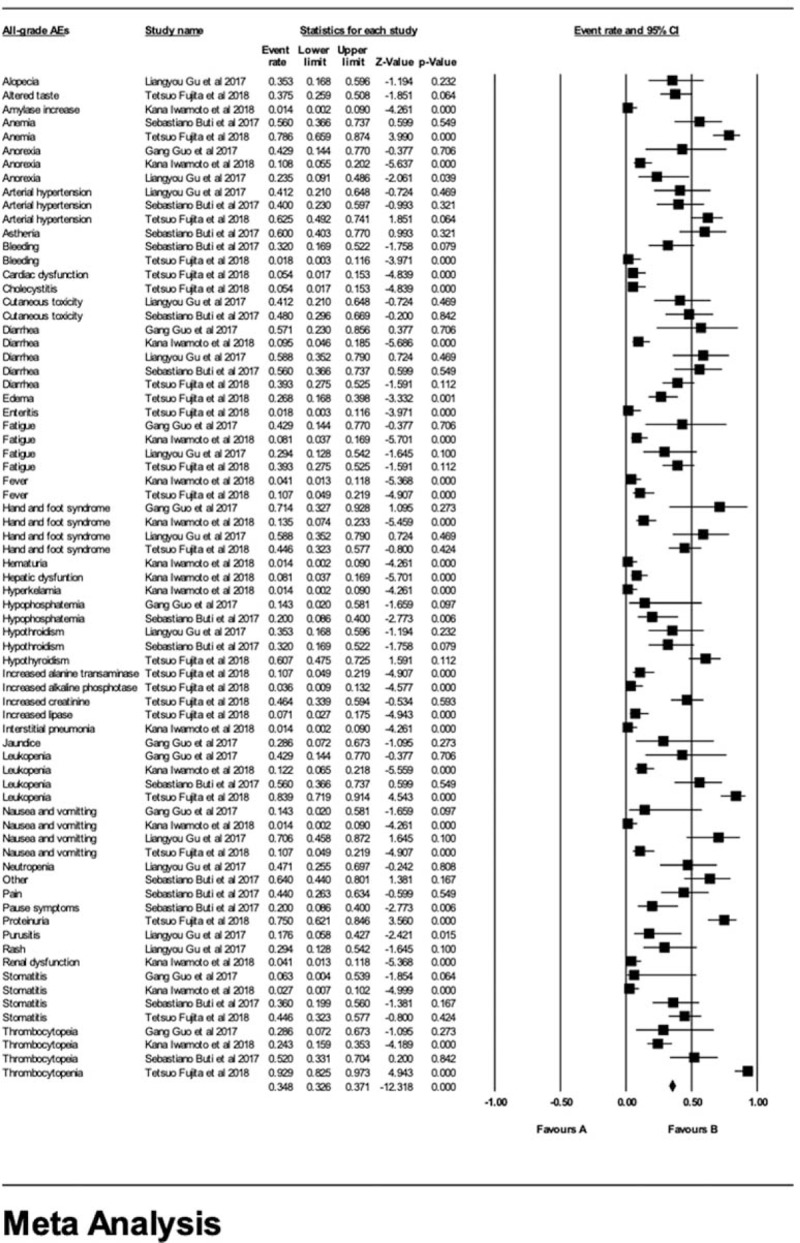
Rates of all-grade AEs in every article.

We also calculated the occurrence rate of all-grade AEs (Fig. 5) in every article we recruited in which anemia, anorexia, arterial hypertension, bleeding, cutaneous toxicity, diarrhea, fatigue, fever, hand and foot syndrome, hypophosphatemia, hypothyroidism, leukopenia, nausea and vomiting, stomatitis, thrombocytopenia were recorded in more than one study. Among these, diarrhea was observed in five studies; fatigue, hand and foot syndrome, leukopenia, nausea and vomiting, stomatitis and thrombocytopenia were observed in four studies; anorexia, arterial hypertension, hypothyroidism were noted in three studies; anemia, bleeding, cutaneous toxicity, fever, hypophosphatemia were recorded in two studies. Other all-grade AEs were recorded only in one study.

### 3.4 Efficacy analysis of the perioperative use of sunitinib

We analyzed the efficacy of the perioperative use of sunitinib in patients with metastatic or advanced RCC. Respectively, we calculated and pooled the parameters reflecting the therapeutic efficiency to both the original and metastatic tumor or tumor thrombus (Table 2A and B). With regard to original tumor, the ORR is 68.8% (44/64). This was much higher than SD and PD rates, which were 17.2% (11/64) and 14.1% (9/64) respectively. Among all studies, seven maintained an ORR over 50%; seven maintained an SD rate less than 20%, and only one had a PD rate over 30%. For the therapeutic efficacy to metastatic tumor or tumor thrombus, the ORR was 78.0% (39/50) compared with SD and PD rates, which were 8.0% (4/50) and 14.0% (7/50) respectively. Generally, the therapeutic efficiency for both the original and metastatic tumor and the tumor thrombus was satisfactory.

**Table 2:**
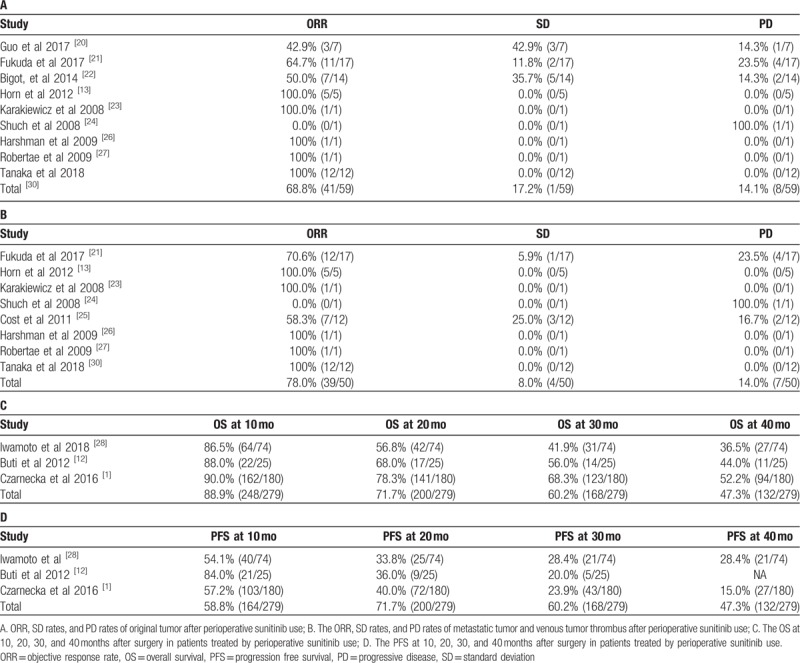
Outcomes of the patients in the included studies.

In addition, we further extracted and pooled the OS and PFS at different time points (Table 2C and D). The pooled OS was 88.9% (248/279) at 10 months, 71.7% (200/279) at 20 months, 60.2% (168/279) at 30months, and 47.3% (132/279) at 40 months. For PFS, the pooled PFS was 58.8% (164/279) at 10 months, 71.7% (200/279) at 20 months, 60.2% (168/279) at 30 months, and 47.3% (132/279) at 40 months.

## 4 Discussion

To the best of our knowledge, this is the first systematic review and meta-analysis evaluating the efficacy and safety of the perioperative appliance of sunitinib in patients with metastatic and advanced RCC. In the safety analysis, our study revealed that proteinuria, anemia, asthenia, pause syndrome, arterial hypertension, and thrombocytopenia were among the most common all-grade AEs, which was consistent with two different studies.^[12,18]^ A closer observation into all-grade AEs also showed that occurrence rate recorded in every single study varied quite tremendously to each other. For example, the pooled occurrence rate of thrombocytopenia was 52.5%, while actually it ranged from 28.6% to 92.9% within four included studies. We noted that the five studies we included to analyze the AEs had quite different compositions of patients, especially the grades of the original tumor, the ages and baseline health conditions of the patients. Consequently, the resulted deviations were caused by unbalanced composition and number of patient samples which led to the wide range of occurrence rate. As for grade ≥3 AEs, arterial hypertension, asthenia, cutaneous toxicity, hypophosphatemia, leukopenia, pain, pause syndrome, renal dysfunction, and thrombocytopenia were the most common types. Accordingly, arterial hypertension and thrombocytopenia occurred frequently after sunitinib intake and maintained a high probability for progress. We believe that the potential interactions between sunitinib and VEGFR and its ligands have contributed to the unstable postoperative blood pressures. Thus, a careful and complete monitoring of blood pressure and platelet count is mandatory in these patients.

Despite unavoidable AEs, we also found high ORRs and SD rate after sunitinib intake. Previously, quite a number of studies have proposed and recommended the perioperative use of sunitinib since improved ORR rates and prolonged OS and PFS had been observed. However, some case reports involving patients of metastatic and advanced RCC especially with intravenous thrombus revealed high degrees of AEs with no obvious improvement in prognostic indicators. By extracting and pooling ORR, SD rate, and PD rate, we primarily confirmed the overall clinical benefit and tumor reduction functions of sunitinib. Since tumor reduction contributes to ease the operation and decreases postoperative modality, our finding could serve as evidence for perioperative, especially preoperative appliance of sunitinib, which could help to obtain the best possible surgical outcome.^[31,32]^ Besides, according to a Phase III study, sunitinib demonstrated satisfactory clinical activity followed by cytokine plus IFN-α treatment in patients with metastatic and advanced RCC, which was deemed as the first-line treatment.^[1,33,34]^ However, the clinical indications to receive second-line treatment and the OS and PFS benefits following first-line treatment should also be further discussed and investigated.^[35]^ With the help of sunitinib, preoperative health conditions could be largely improved, especially the tumorous characteristics. However, researchers found several primary preoperative diseases were negative prognostic factors for perioperative use of sunitinib. These include pretreatment diabetes mellitus, BMI < 25 kg/m^2^ and anemia. Therefore, we are supposed to take care of these conditions before drug intake.^[36,37]^

We still need to confess several limitations of our study. We retrospectively included 14 eligible studies to pool the parameters concerning efficacy and safety of the appliance of sunitinib perioperatively. However, there are three case reports in this meta-analysis and some single center studies failed to engage rational number of patients, both of which may have contributed to potential bias. Meanwhile, the baseline characteristics of patients we included may differ from each other out of our expectation. In addition, the interventions based on sunitinib were not the same between one another in certain articles especially in case reports. Among them, some extreme or personalized intervention were applied, which added to the heterogeneity. Therefore, future studies can pay attention to the underlying bias.

## 5 Conclusion

The most common all-grade AEs led by perioperative use of sunitinib in patients with metastatic and advanced RCC include proteinuria, anemia, asthenia, pause syndrome, arterial hypertension, and thrombocytopenia. And arterial hypertension, asthenia, cutaneous toxicity, hypophosphatemia, leukopenia, pain, pause syndrome, renal dysfunction, and thrombocytopenia are the most common types of grade ≥3 AEs. Meanwhile, in most cases, OS rates, PFS rates turn for the better with the invention of sunitinib. Additionally, ORRs also increase in the original tumor, metastatic sites and tumor thrombus. However, due to the inadequate sample sizes and heterogeneity of the included studies, more clinical researches should be warranted to further evaluate the efficacy and safety.

## Author contributions

**Conceptualization:** Hongyu Jin, Yuying Feng.

**Data curation:** Hongyu Jin.

**Formal analysis:** Jing Zhang.

**Funding acquisition:** Chi Yuan.

**Investigation:** Jing Zhang, Chi Yuan, Yuqi Zhu, Xuelei Ma.

**Methodology:** Chi Yuan, Yuqi Zhu.

**Project administration:** Hongyu Jin, Yuqi Zhu.

**Resources:** Hongyu Jin, Kai Shen, Yuqi Zhu.

**Software:** Jianqi Hao, Yuqi Zhu.

**Validation:** Hongyu Jin, Jianqi Hao, Xuelei Ma.

**Visualization:** Kai Shen.

**Writing – original draft:** Hongyu Jin, Yuying Feng, Xuelei Ma.

**Writing – review & editing:** Hongyu Jin, Yuying Feng, Xuelei Ma.
